# Patients’ and parents’ experiences during wound care of epidermolysis bullosa from a dyadic perspective: a survey study

**DOI:** 10.1186/s13023-022-02462-y

**Published:** 2022-08-13

**Authors:** Petra J. Mauritz, Marieke Bolling, José C. Duipmans, Mariët Hagedoorn

**Affiliations:** 1 Departments of Dermatology, University Medical Center Groningen, University of Groningen, Hanzeplein 1, 9700 RB Groningen, The Netherlands; 2 Department of Health Psychology, HPC FA12, University of Groningen, POB 196, 9700 AD Groningen, The Netherlands

**Keywords:** Wound care, Pain, Anxiety, Parent–child dyad, Epidermolysis bullosa

## Abstract

**Background:**

Epidermolysis bullosa is a rare, often severe, genetic disorder characterized by fragility of the skin and mucous membranes. Despite the important role of parents during wound care, an essential factor in adapting to this disease, studies focusing on the parent–child relationship during wound care are scarce. The current study is aimed at addressing this gap.

**Methods:**

A quantitative study among 31 children (n = 21 ≤ 17 years; n = 10 17–25 years) and 34 parents (including 27 parent–child dyads) was conducted to examine the relationship between pain, itch, anxiety, positive and negative feelings, and coping strategies assessed with the newly developed Epidermolysis Bullosa Wound Care List. The majority of the analyses were descriptive and the results were interpreted qualitatively because of the small sample size.

**Results:**

Children and parents both showed significantly more positive (i.e. ‘protected’, ‘proud’, ‘calm’, ‘connected to each other’ and ‘courageous’) than negative feelings (i.e. ‘helpless’, ‘angry’, ‘insecure’, ‘guilty’, ‘gloomy’ and ‘sad’) during wound care, with parents reporting both feelings more than children. The more children experienced pain, the more they were anxious, had negative feelings, were inclined to use distraction, to postpone wound care and to cry. The more parents experienced feelings (either positive or negative), the more likely they sought distraction. With regard to child-parent dyads the results showed that the more children expressed anxiety, the more parents experienced negative feelings. Furthermore, those who reported more negative feelings were more likely to hide their feelings, while those who reported more positive feelings were more inclined to show their feelings. Pain, itch and anxiety in the child were associated with more distraction or postponement of wound care by the parent.

**Conclusion:**

This study underlines the importance of paying attention to the relationship between feelings and coping strategies in child-parent dyads in the management of pain and anxiety during wound care. Further research could provide more insight how these feelings and coping strategies are related to the psychological well-being of both the child and the parent in the short term as well as in the long term.

## Introduction

Epidermolysis bullosa (EB) comprises a group of rare, often severe, genetic disorders characterized by fragility of the skin and mucous membranes. EB is caused by pathogenic variants in genes encoding for proteins involved in basal epidermal integrity and dermal-epidermal connection [1]. EB can be divided into four main types, depending on the level of blistering. The four major types of EB are EB Simplex (EBS), Dystrophic EB (DEB), consisting of dominant Dystrofic EB (DDEB) and recessive Dystrofic EB (RDEB), Junctional EB (JEB) and Kindler EB [1]. EB can vary greatly in severity by type and subtype. The milder forms of EBS and dominant DEB are often limited to repetitive localized blistering and wounds that can be very painful, while the more severe forms of EBS, recessive DEB and JEB may have generalized blistering and erosions with significant extra-cutaneous involvement, which may cause a lot of pain and itching and require a lot of care. Life expectancy may vary from normal to shortened up to early postnatal death [1, 2].

EB is an example of a pediatric chronic disease that exposes both children and parents to a lot of stress [1–4]. A growing body of evidence endorses the relationship between parental functioning and children’s adaptation to their chronic illness [5, 6]. More parental support, for instance, was linked to lower levels of distress during medical procedures [7] and better psychosocial adjustment in children [8]. Greater parenting stress was associated with poorer psychological adjustment and quality of life in caregivers and children with chronic illness [9] and the parent–child relationship was suggested as an important pathway by which illness might influence symptoms of depression in children with asthma [10].

The importance of parental functioning for chronically ill children also applies to children with EB and their parents, because these parents have a crucial role in managing the disease, especially the wound care. Wound care, generally consisting of popping blisters and caring for or preventing wounds, is one of the challenges that parents and children usually face on a daily basis. Pain and itch are notable characteristics [2, 11–13]. This often is accompanied by (anticipatory) anxiety, which has a significant impact on the quality of life of children with EB [2, 12]. EB can inflict a heavy burden on family members, caused by immense practical and psychological demands that include resource-intensive care and coping with complex feelings [18, 20]. Parents reported pain and anticipatory anxiety of their child, exacerbated by daily care procedures, as two of the main problems they are confronted with [3, 14–16]. Some studies have revealed that parents found it hard to ‘inflict pain’ on their children, which burdened them with negative feelings [1, 17]. Involving the child in wound care, distraction, regulating emotions, encouragement and using rituals were also presented as possible helpful coping strategies to endure wound care [12, 17]. In order to measure diverse aspects of quality of life in EB patients a 17-item questionnaire was developed [18]. However, specific knowledge about the extent to which pain, itch and anxiety or other feelings in the child during wound care is associated with increased anxiety or negative feelings in parents is lacking. In addition, the question arises how coping strategies of children and parents relate to each other.

This study therefore examines the presence of pain, itch, positive and negative feelings, and coping strategies during wound care in both children and their parents. In addition, associations were examined (1) between pain, itch, anxiety and other feelings, (2) between different coping strategies, and (3) between 1 and 2 in children, their parents, and child-parent dyads.

## Materials and methods

### Participants

This study is part of a larger study of parents and children (aged 0–25 years) with EB [19], for which 124 parents and their children were invited. The inclusion criteria were (1) the combination of both a parent and a child (age 7–25) with a genetically confirmed diagnosis of EBS, JEB, RDEB or DDEB, or Kindler EB, and registered as a patient at the Center for Blistering Diseases at the University Medical Centre of Groningen in the Netherlands (UMCG); (2) the parent who performed the wound care most frequently. Children younger than 7 years and their parents were excluded from the current analyses, because these young children were not able to complete the questionnaires themselves. As the population of patients with EB in the Netherlands is small, and parents are usually involved in the care of their child beyond adolescence, children who were young adults were also included (18–25 years).

### Procedure

After the participants had provided their informed consent, they were invited to complete an online questionnaire that was accessible through a secure web portal. If there was more than one child with EB in a family, only the oldest child was included in the study. A reminder mail was sent to participants who had not completed questionnaires after one month.

### Materials

Participants completed the Epidermolysis Bullosa Wound Care List (see additional information for the complete list). This is a self-developed questionnaire about wound care of a child with EB. This questionnaire has been developed, based on a set of 13 interviews [17]. Six parents and seven adult patients with EB have been asked about their experiences with wound care. There are two versions of the list, one for children and one for parents. Children and parents individually completed the list for themselves.

### Pain, itch and anxiety

Pain, itch and anxiety in children were each measured with one item using a scale of six faces, with expressions ranging from very happy (1) to very sad (6). The extreme faces (i.e., very happy and very sad) were labelled with “no pain/itch/anxiety at all” and “a lot of pain/itch/anxiety.” Parents completed only the anxiety item.

### Positive and negative feelings

Negative and positive feelings in children and parents were assessed with 5 and 6 items, respectively. Negative feelings included ‘helpless’, ‘angry’, ‘insecure’, ‘guilty’, ‘gloomy’ and ‘sad’. The positive feelings were ‘protected’, ‘proud’, ‘calm’, ‘connected to my parents (my child for parents)’ and ‘brave (courageous for parents)’. The items were scored on 4-point scales, ranging from ‘not at all’ (1) to ‘very much’ (4) and averaged into one score for negative (Cronbach's alpha = 0.75 for children and 0.86 for parents) and positive feelings (Cronbach's alpha = 0.82 for children and 0.82 for parents).

### Coping strategies

Both children and parents reported their coping strategies, including distraction, collaboration, and postponing wound care, crying (children)/expressing emotions (parents), and becoming silent (children)/hiding emotions (parents). These strategies were scored on a 4-point scale ranging from ‘not at all’ (1) to ‘very much’ (4). All strategies were assessed with one item, except ‘hiding emotions’ in parents that was assessed with three items (i.e., ‘I am focussing on different things’, ‘I am disconnecting from my feelings’, and ‘I am hiding my feelings’; Cronbach’s alpha = 0.80).

### Data analysis

All analyses were done using SPSS version 25. The majority of the analyses were descriptive and results were interpreted qualitatively due to the small sample size. First, the means and standard deviations were calculated for pain, itch, different feelings and coping strategies of children with EB and their parents. Next, Spearman correlations were used as the sample sizes consisted of around 30 participants. There were some missing values in the questionnaires for a number of the children and the parents, resulting in small differences in sample sizes for the different analyses.

## Results

The current study included data of 31 children and young adults (16 boys and 15 girls, 21 ≤ 17 years; 10 between 17 and 25 years, 17EBS; 3 JEB; 4 DDEB and 4 RDEB; 2 Kindler Syndrome, 1 subtype missing), and 34 parents (25 women and 9 men) who formed 27 parent–child dyads (both members of the dyad same subtype). The average age of the parents was 43 years (ranging between 36 and 64 years). One dyad was of Middle Eastern ethnicity and the rest of European ethnicity. Most children (25) reported wound care took less than an hour per day, while 5 children (1 EBS; 1 JEB; 3 RDEB) indicated more than an hour per day. Reasons for invitees’ nonparticipation in the study were as follows: no reason given (68%), incorrect address (13%), insufficient personal benefits from participation (15%), an intellectual disability (2%) and incorrect inclusion (2%). Wound care consisted of (in descending order of frequency) popping blisters, putting on new wound dressings, removing wound dressings, nail care, anointing the wounds and skin with cream or oil, removing wound crusts, bathing child, showering child, cleaning wounds without shower or bath, callus care and taking care of a probe.

### Pain, itch, feelings and coping strategies during wound care in children and parents

On average, children of all subtypes did not score high on pain, itch and anxiety (i.e., below or little above the mid-point of the scale), and children and parents showed similar scores for anxiety with no significant difference between them (See Table 1). In addition, children and parents showed agreement on the use of the coping strategies ‘help from child’ and ‘postponing wound care’. Although children mentioned the use of ‘distraction’ more often than did their parents, there were no significant differences in scores. Children and parents both showed significantly more positive than negative feelings. Of note, children as well as their parents were more likely to become silent or hide their emotions than to show their emotions, but this difference was only significant in children. There were no notable differences in pain, itch and anxiety, feelings and coping strategies scores per EB subtype.

**Table 1:** Descriptives statistics pain, itch, feelings and coping strategies of children and parents during wound care

	Children (N = 31)					Parents (N = 34)				Paired *t*-test	
	Min	Max	Mean	SD		Min	Max	Mean	SD	t	*p*
Pain	1	6	2.68	1.47							
Itch	1	6	2.16	1.16							
Anxiety	1	4	1.58	.96		1	5	1.71	1.06	0.64	0.52
Positive feelings	1	3.80	2.05	0.79		1	4	2.56	0.81	2.13	0.04
Negative feelings	1	2.67	1.38	0.44		1	3.33	1.93	0.68	5.89	0.00
Distraction	1	4	2.03	1.10		1	4	1.76	0.86	− 1.94	0.06
Help from child	1	4	2.90	1.19		1	4	2.88	1.12	− 0.16	0.87
Postponing wound care	1	4	1.48	0.77		1	3	1.58	0.61	0.85	0.40
Crying/showing emotions	1	3	1.35	0.61		1	4	1.76	0.87	2.2	0.04
Becoming silent/hiding emotions	1	4	2.26	1.09		1	4	1.96	0.91	− 0.74	0.47

	Children (N = 31)					Children (N = 31)					
	Min	Max	Mean	SD		Min	Max	Mean	SD	t	*p*
Positive feelings	1	3.80	2.05	0.79	Negative feelings	1	2.67	1.38	0.44	4.61	0.00
Crying	1	3	1.35	0.61	Becoming silent	1	4	2.26	1.09	− 4.12	0.00

	Parents (N = 34)					Parents (N = 34)					
Positive feelings	1	4	2.56	0.81	Negative feelings	1	3.33	1.93	0.68	4.61	0.00
Showing emotions	1	4	1.76	0.87	Hiding emotions	1	4	1.96	.91	− 0.86	0.40

N = sample; Min = minimum; Max = maximum; SD = standard deviation

### Relationship between pain, itch and feelings in children and parents, and child-parent dyads

In contrast to itch, pain was strongly related to anxiety and negative feelings in children. Positive feelings were not significantly related to pain, itch, anxiety. In parents, anxiety and other negative feelings were strongly related. With regard to child-parent dyads, the results showed that children who expressed more anxiety had parents who experienced more negative feelings (see Table 2).

**Table 2:** Spearman correlations (and *p*-values) between pain, itch, and feelings within children, parents and child-parent dyads

	Pain	Itch	Anxiety child	Positive feelings child	Negative feelings child	Anxiety parent	Positive feelings parent	Negative feelings parent
Pain	1	0.26 (0.15)	0.62** (< 0.001)	0.04 (0.81)	0.37* (0.04)	0.12 (0.60)	− 0.23 (0.25)	0.36 (0.06)
Itch		1	0.04 (0.85)	0.30 (0.10)	0.12 (0.54)	− 0.08 (0.71)	− 0.08 (0.69)	0.16 (0.43)
Anxiety child			1	0.17 (0.35)	0.28 (0.13)	0.31 (0.12)	0.09 (0.65)	0.47* (0.01)
Positive feelings child				1	0.26 (0.16)	− 0.13 (0.54)	0.26 (0.24)	0.00 (0.98)
Negative feelings child					1	0.12 (0.55)	− 0.051 (0.80)	0.11 (0.58)
Anxiety parent						1	0.11 (0.54)	0.52** (< 0.001)
Positive feelings parent							1	0.29 (0.10)
Negative feelings parent								1

Children (N = 31), Parents (N = 34), Dyads (N = 27)

^*^p < 0.05 (2-tailed)

^**^p < 0.01

### Associations between coping strategies within children, parents and child-parent dyads

In children, the results showed positive correlations of postponing wound care with distraction and crying. Parents’ coping strategies were not significantly related to each other. In dyads, only the coping strategies ‘distraction’ and ‘help from child’ were strongly linked to each other while the remaining coping strategies showed no mutual relationship (see Table 3).

**Table 3:** Correlations (Spearman) between coping strategies within children, parents and dyads

	Distraction child	Help from child/child	Postponing woundc care child	Crying	Becoming silent	Distraction parent	Help from child/parent	Postponing wound care parent	Showing emotions	Hiding emotions
Distraction child	1	− 0.16 (0.39)	0.38* (0.03)	0.35 (0.05)	0.17 (0.37)	0.38* (0.48)	− 0.06 (0.78)	0.06 (0.78)	0.00 (0.99)	0.16 (0.43)
Help from child/c		1	0.10 (0.61)	0.01 (0.95)	0.26 (0.16)	− 0.17 (0.40)	0.50** (< 0.001)	− 0.03 (0.87)	− 0.04 (0.89)	0.04 (0.84)
Postponing wound care. child			1	0.53** (< 0.001)	0.29 (0.12)	0.12 (0.56)	0.02 (0.93)	0.27 (0.18)	0.18 (0.37)	0.07 (0.72)
Crying				1	0.14 (0.47)	− 0.03 (0.87)	0.2 (0.33)	0.28 (0.16)	0.34 (0.09)	0.31 (0.12)
Becoming silent					1	− 0.11 (0.58)	0.14 (0.49)	0.03 (0.90)	− 0.01 (0.96)	0.03 (0.89)
Distraction parent						1	− 0.28 (0.12)	0.29 (0.11)	− 0.17 (0.34)	0.34 (0.06)
Help from child/parent							1	− 0.07 (0.68)	− 0.07 (0.62)	0.02 (0.90)
Postponing wound care parent								1	0.04 (0.85)	0.11 (0.54)
Showing emotions									1	− 0.06 (0.68)
Hiding emotions										1

Children (N = 31), Parents (N = 34), Dyads (N = 27)

^*^p < 0.05 (2-tailed)

^**^p < 0.01

### Associations between feelings and coping strategies within children, parents and child-parent dyads

Children who experienced more pain tended to distract their attention from the wound care, asked for postponement of the wound care and cried more than children who experienced less pain. Furthermore, children cried more when they experienced more anxiety or other negative feelings. Parents who experienced more negative feelings were more inclined to distract their child during wound care, to postpone the wound care and to hide their emotions. Furthermore, parents who experienced more positive feelings were more likely to distract their child and show their emotions. Pain, itch and anxiety in the child were also associated with more distraction and postponement of wound care by the parent. On the other hand, anxiety and other negative and positive feelings of parents were not linked to coping strategies of children (see Table 4).

**Table 4:** Correlations (Spearman) between feeling s and coping strategies within children, parents and dyads

	Children					Parents				
	Distraction child	Help from child	Postponing woundc care child	Crying	Becoming silent	Distraction parent	Help from child	Postponing wound care parent	Showing emotions	Hiding emotions
Pain	0.46** (< 0.001)	0.01 (0.95)	0.60** (< 0.001)	0.54** (< 0.001)	0.19 (0.31)	0.38* (0.04)	− 0.07 (0.72)	0.42* (0.03)	0.19 (0.34)	0.32 (0.11)
Itch	0.13 (0.48)	− 0.08 (0.66)	0.03 (0.87)	− 0.02 (0.92)	0.08 (0.68)	0.47* (0.01)	− 0.36 (0.07)	− 0.034 (0.87)	− 0.05 (0.81)	0.27 (0.17)
Anxiety child	0.30 (0.10)	− 0.24 (0.20)	0.35 (0.05)	0.53** (< 0.001)	− 0.01 (0.97)	0.42* (0.03)	− 0.08 (0.68)	0.50** (< 0.001)	0.28 (0.16)	0.17 (0.39)
Negative feelings child	0.17 (0.35)	− 0.05 (0.80)	0.29 (0.11)	0.78** (< 0.001)	0.15 (0.42)	− 0.11 (0.58)	0.21 (0.28)	0.14 (0.49)	0.36 (0.06)	0.21 (0.31)
Positive feelings child	0.26 (0.16)	− 0.37* (0.04)	− 0.07 (0.70)	0.13 (0.49)	0.09 (0.63)	0.16 (0.42)	0.00 (0.98)	− 0.08 (0.69)	0.09 (0.67)	− 0.12 (0.54)
Anxiety parents	− 0.14 (0.48)	0.15 (0.47)	0.04 (0.85)	0.15 (0.47)	− 0.01 (0.97)	0.17 (0.34)	0.14 (0.42)	0.20 (0.26)	0.23 (0.18)	0.19 (0.29)
Negative feelings parent	0.22 (0.27)	− 0.05 (0.81)	0.24 (0.23)	0.13 (0.43)	0.14 (0.49)	0.64** (< 0.001)	0.04 (0.82)	0.39* (0.02)	− 0.02 (0.91)	0.49** (< 0.001)
Positive feelings parent	− 0.17 (0.41)	− 0.11 (0.58)	0.16 (0.41)	0.09 (0.67)	− 0.20 (0.31)	0.35* (0.04)	0.11 (0.54)	0.24 (0.19)	− 0.28 (0.11)	0.11 (0.54)

Children (N = 31), Parents (N = 34), Dyads (N = 27)

^*^p < 0.05 (2-tailed)

^**^p < 0.01

## Discussion

Our findings illustrate the associations between feelings and coping strategies of children with EB and their parents. However, there are no notable differences in scores with regard to pain, itch, anxiety, other feelings en coping strategies per EB subtype. Moreover the numbers are too small and standard deviations too big to draw any conclusions on differences in scores per EB subtypes. Children and parents both showed significantly more positive than negative emotions during wound care. Pain and anxiety in children were associated with negative feelings and various coping strategies in both children and parents.

### Pain, itch, anxiety and feelings

The minor differences in pain, itch and anxiety scores per EB subtype in this study are not clearly endorsed by the results of other studies. Some studies have shown that patients with RDEB experienced worsened quality of life, decreased functioning and social activities, and increased pain and itch when compared to other EB subtypes [3, 20, 21] while another review has revealed quality of life was more affected in people who have RDEB and JEB [22]. The present study underlines previous results that pain due to wound care is strongly linked to anxiety in children with EB [2, 12, 14, 23]. Anxiety in children appeared to be closely related to negative feelings (e.g. angry, guilty and sad) in their parents, which probably partly explains why parents experience caring for a child with EB as very burdensome [1, 16, 20, 24, 25]. It is conceivable that a vicious circle exists in which feelings of one member of the child-parent dyad reinforce or maintain feelings in the other member. To the best of our knowledge it has not been reported in earlier studies that children with EB and parents reported more positive than negative feelings during wound care. More positive feelings in both were not associated with less anxiety or lower degree of negative feelings in children or parents. This is conceivable as previous studies of the structure of feelings have shown that positive and negative feelings have consistently emerged as two dominant and relatively independent dimensions [26].

### Coping strategies

The outcome that children and parents seek distraction or postpone wound care to cope with pain or negative feelings is in accordance with other studies focusing on psychosocial aspects of wound care in patients with EB [12, 17, 23, 27]. Distraction seems to be an important strategy for parents, which is associated with their child's pain, itch and anxiety, but also with a wide range of feelings they perceive themselves suggesting it is helpful for them both.

It appears that ‘becoming silent’ or ‘hiding emotions’ were relatively more common than expressing emotions. It is noteworthy that crying in children is strongly linked to their pain, anxiety and negative feelings, which suggests that crying is an important indicator of the child's physical well-being. The tendency of parents to hide their emotions during wound care corresponds with earlier studies where it is suggested as a strategy to be able to fulfill their role as caregiver [3, 17]. These results possibly indicate that parents do not want to burden their children more than necessary or do not give attention to their emotions to be able to perform the wound care. Furthermore, it emerged that the child's help was clearly present in the wound care. In previous studies the involvement of the child in wound care has been seen as an important strategy to endure wound care [12], however the results of this study did not reveal any relationship between which strengthens this.

The different roles of children and parents in wound care might be an important perspective that partly can be the explanation for no relationship between parental feelings and children’s coping strategies. Children are at various stages of development and the recipient of care, which may leave them unable to focus on their parents' feelings. In addition, the fact that parents hide their emotions prevents children to respond to them.

### Strengths and limitations of the study

The strength of our study is that all children and adolescents with EB > 7 years up to 25 years and their parents were approached in the Netherlands, and a reasonable response rate of 21% was obtained. However, the limited sample size and the heterogeneity of EB-types may have affected the generalizability of the findings to the whole group of EB patients, so that the results should be interpreted cautiously. Moreover, the cross-sectional design does not warrant any causal conclusions. It still remains unclear what the causality of the relationship is between feelings and coping strategies during wound care, and how they are related to the psychological well-being of both the child and the parent. Additional research could provide more insight into this. Further the psychometric characteristics of the EB Wound Care List have not yet been assessed, whereby no statements could be made about the validity of the questionnaire At the same time this list is already being used in clinical practice to start a discussion about wound care during consultation hours, especially when high scores for pain, itch, anxiety, or negative feelings are reported. In that respect, it meets a need and could be seen as a first step towards the further development of a validated list. The limitations of this study require a little more nuance. The study concerns a relatively small population of patients with complicated and severe symptoms, of which approximately one fifth participated nevertheless. In addition, this is one of the first quantitative studies that highlight the relationship between children with EB and parents during a complex task for both of them. Finally, the results of this study emphasize that attention to pain and anxiety in the child during wound care should be prioritized in both research and clinical practice from dyadic perspective because it is conceivable that both could threaten children’s adaptation to their EB and undermine their and their parents’ well-being.

## Conclusions

The current study shows the importance of attention to parental functioning, feelings and coping strategies during wound care, given the dyadic processes between children’s and parental feelings and coping strategies. In addition, it seems relevant to pay attention to hidden emotions of parents that possibly are an indicator of the burden of wound care, as parents tend to hide their feelings when they are negative. Finally, the more a child shows pain and anxiety, the more important it is to support the involved parents early as possible given the relationship with increased negative feelings in them.

## Data Availability

The data and materials can be requested from the corresponding author.

## Abbreviations

DEB: Dystrophic epidermolysis bullosa
DEBRA: Dystrophic Epidermolysis Bullosa Research Association
EB: Epidermolysis bullosa
EBS: Epidermolysis bullosa simplex
HRQoL: Health-Related Quality of Life
JEB: Junctional epidermolysis bullosa
KS: Kindler syndrome
RDEB: Recessive dystrophic epidermolysis bullosa
